# An Evaluation of Total Internal Motions of Locally Advanced Pancreatic Cancer during SABR Using Calypso^®^ Extracranial Tracking, and Its Possible Clinical Impact on Motion Management

**DOI:** 10.3390/curroncol28060389

**Published:** 2021-11-11

**Authors:** Hrvoje Kaučić, Domagoj Kosmina, Dragan Schwarz, Adlan Čehobašić, Vanda Leipold, Ivo Pedišić, Mihaela Mlinarić, Matea Lekić, Hrvoje Šobat, Andreas Mack

**Affiliations:** 1 Specijalna Bolnica, Radiochirurgia Zagreb, Franje Tuđmana 4, 10431 Sveta Nedelja, Croatia; domagoj.kosmina@radiochirurgia.hr (D.K.); dragan.schwarz@radiochirurgia.hr (D.S.); adlan.cehobasic@radiochirurgia.hr (A.Č.); vanda.leipold@radiochirurgia.hr (V.L.); ivo.pedisic@radiochirurgia.hr (I.P.); mihaela.mlinaric@radiochirurgia.hr (M.M.); matea.lekic@radiochirurgia.hr (M.L.); hrvoje.sobat@radiochirurgia.hr (H.Š.); 2 Sveučilište Josipa Jurja Strossmayera u Osijeku, Medicinski Fakultet Osijek, Josipa Huttlera 4, 31000 Osijek, Croatia; 3 Medicinski Fakultet Sveučilišta u Rijeci, Braće Branchetta 20/1, 51000 Rijeka, Croatia; 4 Swiss NeuroRadiosurgery Center, Bürglistrasse 29, 8002 Zürich, Switzerland; a.mack@snrc.ch

**Keywords:** Calypso^®^ extracranial tracking, dose escalation, LAPC, motion management, pancreatic SABR

## Abstract

(1) Background: the aims of this study were to determine the total extent of pancreatic cancer’s internal motions, using Calypso^®^ extracranial tracking, and to indicate possible clinical advantages of continuous intrafractional fiducial-based tumor motion tracking during SABR. (2) Methods: thirty-four patients were treated with SABR for LAPC using Calypso^®^ for motion management. Planning MSCTs in FB and DBH, and 4D-CTs were performed. Using data from Calypso^®^ and 4D-CTs, the movements of the lesions in the CC, AP and LR directions, as well as the volumes of the 4D-CT-based ITV and the volumes of the Calypso^®^-based ITV were compared. (3) Results: significantly larger medians of tumor excursions were found with Calypso^®^ than with 4D-CT: CC: 29 mm (*p* < 0.001); AP: 14 mm (*p* < 0.001) and LR: 11 mm (*p* < 0.039). The median volume of the Calypso^®^-based ITV was significantly larger than that of the 4D-CT based ITV (*p* < 0.001). (4) Conclusion: beside known respiratory-induced internal motions, pancreatic cancer seems to have significant additional motions which should be considered during respiratory motion management. Only direct and continuous intrafractional fiducial-based motion tracking seems to provide complete coverage of the target lesion with the prescribed isodose, which could allow for safe tumor dose escalation.

## 1. Introduction

Patients with unresectable, locally advanced pancreatic cancer (LAPC) still represent one of the most challenging subset of patients in oncology. The 5-year overall survival (OS) rate is approximately 7–10%, and the median survival of resected patients following adjuvant therapy ranges from 20 to 28 months, leading to a specific 5-year survival rate around 10–20% [1]. Locoregional failure affects 50–80% and systemic relapse over 70% of patients, respectively [2]. A significant proportion of patients with LAPC, especially those with poor performance status, are still treated as patients with distant disease [3]. The addition of radiochemotherapy can be beneficial, but its role in the course of treatment still remains unclear [4]. Stereotactic ablative radiotherapy (SABR) has emerged as an effective and safe form of local treatment for patients with LAPC in recent years, but, similar to chemoirradiation, the value of SABR in terms of prognosis is unclear [4,5,6,7,8].

The main clinical advantage of SABR is its high ablative dose delivery with extreme hypofractionation, as well as the precision and accuracy provided by the stereotactic technique. Steep dose falloff around the target volume and motion management are the next-most important features of SABR. Motion management plays a crucial role in sparing surrounding organs at risk (OARs), which are the major limiting factor for dose escalation. The organs adjacent to the pancreas, the duodenum, stomach and small bowel, are highly radiosensitive, and it is critical to spare them from any excess of radiation [9,10,11]. Hoyer et al. demonstrated high gastrointestinal toxicity (grade ≥ 3 in 44% patients) caused by SABR using considerable dose escalation with a biological effective dose (BED) of 112 Gy to large target volumes without motion management of the lesion and no adequate OAR sparing [12].

Movements of the pancreas are unpredictable, and it seems that there are, beside respiratory, other significantly influencing motions, such as peristaltic, heartbeat induced, changes of the breathing pattern (frequency, amplitude), stomach and intestine filling, sudden, patient induced (coughing, stirring...), and probably some unknown. The flatulence often caused by diet and patient anxiety also has a strong effect on the daily anatomical variations of a pancreatic tumor’s position and movements. Furthermore, daily CBCT kilovoltage imaging of abdominal regions has low contrast, especially in free breathing (FB) patients, which also complicates image guidance. Bone matching is generally unreliable, soft tissue matching is often almost impossible, and only fiducial matching remains as reliable image guidance [13]. The primary intention of SABR is to escalate the dose to the target volume and consequently improve the local control, as shown by Brunner et al. [11]. There are several ways to deal with the tumor movement in the abdomen during SABR, and they can be basically divided into two groups: motion mitigation and motion tracking techniques. Motion mitigation is typically achieved with abdominal compression to reduce respiratory motions, using commercially available devices. This technique is generally well tolerated by the vast majority of patients, but for some of them it can be unpleasant and painful. The main drawback of abdominal compression is pushing the OARs closer to the target volume. Deep breath hold (DBH) during SABR is also a possible motion mitigation technique, but without adequate monitoring, it is not sufficiently accurate [14]. Motion management can be achieved via respiratory gating realized through 4D-CT prospective planning, using the tumor’s position in the selected optimal phase of breathing cycle as a gross tumor volume (GTV)/clinical target volume (CTV). Respiratory gating relies on the motion tracking of an external surrogate that correlates with the tumor’s motion. The main drawback of respiratory gating is prolongation of the treatment time, especially with patients that do not breathe consistently. As shown by Campbell et al., the correlation between an external surrogate and the internal target only accounts for respiratory motion and it would most likely benefit from continuous direct target tracking [15]. Retrospective 4D-CT planning, using all phases of 4D-CT studies for the reconstruction and generation of the internal tumor volume (ITV), is another technique to compensate for tumor motion. The main drawback of this motion management technique is generation of a large target volume, that inevitably invades the OARs, but the technique itself is generally fast and pleasant for the patient. Moreover, previous studies have demonstrated significant discrepancies between the abdominal target position observed by planning 4D-CT and the target position observed by the daily CBCT for the same patients at the treatment table [16,17,18,19]. Those discrepancies suggest that more comprehensive techniques for motion evaluation are needed [20]. Intrafractional management of tumor motions during the dose delivery can be either fiducial-based or cine MRI-based. Fiducial-based intrafractional motion management uses radio-opaque fiducials (e.g., made of gold) implanted into or nearby the tumor, that are tracked with kilovoltage imaging during dose delivery. The technique is generally fast and reliable, and most often is used in robotic arm based linacs, but it can lead to the additional patient exposure to ionizing radiation. MR-linacs use on-board cine MRI devices for the intrafractional motion management of soft tissue during dose delivery. The technique is non-invasive and very accurate, but some gating uncertainties still remain due to the time delay of the MRI.

This study was primarily designed to investigate and determine the full extent of movements of pancreatic cancers in the cranial–caudal (CC), anterior–posterior (AP), and left–right lateral (LR) directions during SABR, using the data of tumor’s motions recorded with Calypso^®^ extracranial tracking during SABR of LAPC for 34 patients. For comparison, we used the data of tumor’s motions recorded during 4D-CT planning for the same patients. By default, 4D-CT predominantly presents tumor’s respiratory movements, with minimal impact of other movements. We assume that our data from Calypso^®^ will reveal significant additional pancreatic cancer’s movements – most likely caused by peristalsis, heartbeat, changes of the breathing pattern (frequency, amplitude), stomach and intestine filling, drift, and possibly some other still undetermined. We aimed also to investigate the level of the additional movements of those pancreatic tumors, and to indicate possible clinical advantages of a real time continuous intrafractional motion management of all pancreatic tumor’s movements. These findings could reveal the practical imperfections of motion management techniques that predominantly mitigate or track respiratory movements (i.e., abdominal compression, deep breath hold and respiratory gating).

Unlike other, commonly used tumor-implanted fiducials that are tracked radiographically, the Calypso^®^ extracranial tracking system uses fiducial transponders consisting of a glass envelope which contains metal coil. The fiducials are tracked electromagnetically by the array using non-ionizing radio frequencies in real time, with 20 Hz frequency and submillimeter accuracy.

## 2. Patients and Methods

### 2.1. Patients

Treatment planning data of thirty four patients (23 male and 11 female) with locally advanced pancreatic cancer treated with SABR using Calypso^®^ extracranial tracking for intrafractional motion management were analyzed in this retrospective, single-arm, and single-institution observational study. All patients were discussed and approved by the institution’s multidisciplinary tumor board prior to SABR. Inclusion criteria were: histologically proven pancreatic cancer, age ≥ 18 years, ECOG 0–2, radiologically negative regional lymph nodes and no distant metastasis, no previous abdominal radiotherapy, no radiological signs of gastric or duodenal obstruction, and no concurrent chemotherapy. LAPC was defined as unresectable, histologically proven pancreatic adenocarcinoma or islet cell carcinoma (neuroendocrine carcinoma-NEC) of the pancreas. Resectability was assessed by the institution’s multidisciplinary tumor board, according to the arterial and venous criteria for resectability status primarily [3].

Patients’ mean age was 67 (ranging 45–87); 25 patients (74%) had cancer located in the pancreatic head and 9 patients (26%) had cancer located in pancreatic body or tail. Thirty (88%) patients had pancreatic adenocarcinoma and four (12%) patients had pancreatic islet cell carcinoma. All patients underwent a MSCT-guided implantation of minimal two or optimal three Calypso Beacon^®^ transponders into the tumor, at least fourteen days before treatment planning. MRI scans in DBH were done on the same day, prior to implantation, to avoid the notable artefacts that transponders cause on the MRI scans. All clinical procedures performed were in accordance with the national medical ethical standards and with the 1964 Helsinki declaration and its later amendments, or comparable ethical standards. Informed consent was signed by, and obtained from every patient.

### 2.2. Stereotactic Ablative Radiotherapy

A contrast-free multi slice computed tomography (MSCT) scan in FB, DBH as well as 4D-CT study sets, with a slice thickness of 1 mm and pre-implantation contrast-free MRI in DBH of the abdomen (T1 and T2) were acquired for all patients, and subsequently coregistered (with deformable registration methods). The CTV was defined as the GTV with no additional margins.

Treatments were planned in two ways:

1. For the patients treated in FB, the phase gated 4D-CT was used and coregistered with MRI. CTV was delineated on the T1 or T2 images of the MRI, and further corrected on gated 4D-CT scan, as needed.
2. For the patients treated in DBH, the MSCT in DBH was used and coregistered with MRI. CTV was delineated on the T1 or T2 images of the MRI, and further corrected on MSCT in DBH scan, as needed.

Patients were in supine position on either a wing-board or vacuum pillow, with the arms above the head during the treatment. No immobilization was used. Treatments were delivered by a Varian EDGE^®^ linear accelerator (Varian Medical Systems, Palo Alto, CA, USA). SABR plans were optimized and delivered using multiple coplanar arcs (Volumetric Arc Therapy-VMAT), or multiple noncoplanar intensity modulated radiotherapy (IMRT) sliding window fields. Beam energy of 6 and/or 10 MV with unflattened, flattening filter free (FFF) photon beams was used for all patients. Dose delivery technique and beam energies were chosen dependent on patient anatomy and fractionation schemes to achieve best dose distributions while having plans with low modulation and high QA passing rates.

Biological effective dose was calculated using alpha/beta = 10 Gy (BED_10_). A median BED_10_ of 112.5 Gy (ranging from 85.5–128.9 Gy) in 1, 3 or 5 consecutive daily fractions (9–31.25 Gy per fraction) was applied with a normalization of 80% of the prescribed dose to 98% to 99.5% of the planning target volume (PTV). PTV was generated using 3 mm margin to CTV for all patients. Fractionation regimes and corresponding BED_10_ for each were: 5 × 9 Gy (BED_10_ = 85.5 Gy), 3 × 15 Gy (BED_10_ = 112.5 Gy) and 1 × 31.25 Gy (BED_10_ = 128.9 Gy).

The optimal fractionation for each patient individually was chosen to achieve the goal of OARs sparing. The primary OARs for pancreas targets were the stomach, duodenum, and the small intestine. For a single fraction treatment we used Dmax V(0.03 ccm) < 23 Gy, V(20 Gy) < 3.3 ccm and V(15 Gy) < 9.1 ccm, for 3 fraction treatment we used V(1 ccm) < 31.4 Gy, V(5 ccm) < 23.2 Gy and V(10 ccm) < 16.7 Gy while for 5 fraction treatment we used V(1 ccm) < 36 Gy, V(5 ccm) < 25.5 Gy and V(10 ccm) < 18.5 Gy, for all primary OARs [21]. We followed RTOG recommendations for the dose–volume constraints for all other OARs [22]. The dose was applied extremely heterogeneously. The mean dose to the PTV was higher than the prescription dose, and there was no planning constraint on the maximum dose as long as it was located inside the PTV. Image guidance was performed daily before each fraction, by registering cone beam CT imaging with the planning MSCT, primarily using Calypso^®^ transponders for registering, to verify the correct position of the patient and the lesion. Prior to beam on, the Calypso^®^ system initially checked that the position of the transponders represented the planned transponder positions using an electromagnetic array, and a geometric deviation of 2 mm or less and/or a rotation of 20 degrees or less were acceptable. The final treatment position was determined using CBCT, matching soft tissue, bony anatomy and transponders, with primacy given to the transponder’s position. During the treatment, we used a 2 mm gating window, i.e., the transponders were allowed to move 2 mm in any direction from the treatment position before the beam was shut off.

### 2.3. Calypso^®^ Extracranial Tracking

**Figure 1:**
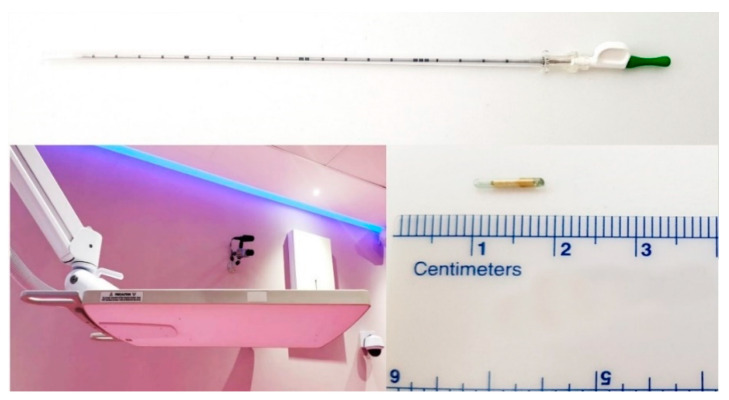
Implantation needle; electromagnetic array; Beacon^®^ transponders.

Calypso^®^ extracranial tracking (Varian Medical Systems, Palo Alto, CA, USA) is a real time intrafractional motion management system, FDA approved for motion management of soft tissue tumor lesions [23,24]. The Calypso^®^ extracranial tracking system consists of Calypso Beacon^®^ transponders: radio frequency electromagnetic fiducials consisting of a glass envelope which a contains metal coil, and an electromagnetic array (Figure 1). The electromagnetic array detects the position and movements of each of the transponders implanted into the tumor in three axes (longitudinal, horizontal and sagittal) and in three plains (transversal, coronal and sagittal). The system provides the three-dimensional intrafractional motion management of all movements of the lesion in real time with 20 Hz frequency and submillimeter accuracy. The transponders are hypoallergenic and non-toxic. The Calypso^®^ system uses non-ionizing radio frequencies to localize the transponder. At least two Beacon^®^ transponders are needed for the Calypso^®^ system to detect linear movements, and three transponders are needed to detect rotational movements. Beacon^®^ transponders were percutaneously implanted into or adjacent to the lesion by an educated and skilled interventional radiologist in our institution under CT-guidance in local anesthesia, using the implantation needle provided by the manufacturer (Figure 1).

**Figure 2:**
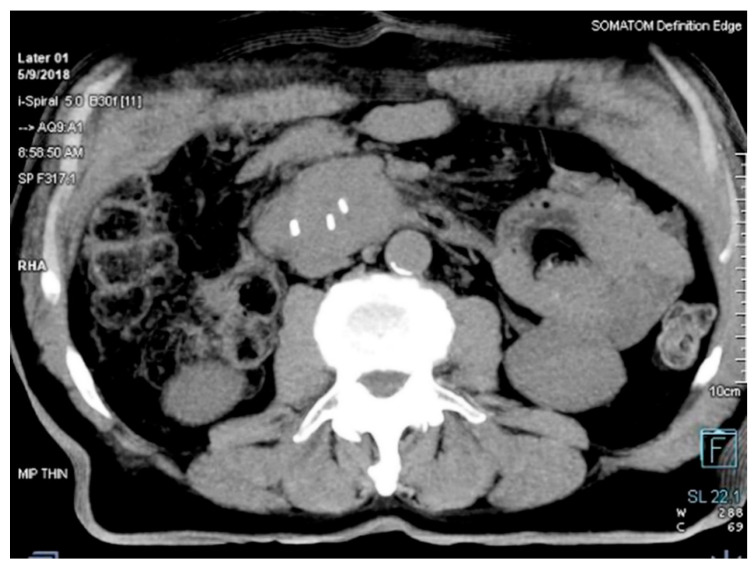
Position of the Beacon^®^ transponders in the tumor on a MSCT scan.

The distance between transponders in the lesion was minimum 1 cm, and maximum 7 cm, according to the manufacturer’s manual, to provide accurate motion tracking (Figure 2).

The transponders were implanted at least fourteen days prior to treatment planning, to allow their in-site stabilization, to prevent possible migrations during the periods between implantation, planning, and treatment, and also to prevent such migrations during the treatment. On the day of the implantation, every patient remained in our institution for eight-hour time due to the observation and routine blood work needed to test for possible internal bleeding. Contraindications for Beacon^®^ transponder implantation were coagulopathies, neuromuscular diseases, acute infection disease and general contraindications for contrast-enhanced CT scans, as well as unfavorable patient anatomy in the abdomen, according to the interventional radiologist’s evaluation. There were no complications or side effects noticed during or after the implantation of the fiducials.

### 2.4. Tumor Excursions Measurements–4D-CT

All measurements of tumor motion in the CC, AP and LR directions were made with the Eclipse^®^ (Varian Medical Systems, Palo Alto, CA, USA) planning system, using the data collected from planning 4D-CT scans.

We defined the GTV as the position and anatomy of the tumor on the phase-gated 4D-CT scan in the 20% inhale phase, and then generated corresponding ITV in two different ways:

1. When the tumor contours were clearly recognizable on reconstructed maximum intensity projection (MIP) CT scans, we used MIP-based ITV for each patient.
2. When tumor contours could not be clearly differentiated on MIP, we deformably propagated the GTV contour to other 4D-CT breathing phases, made manual corrections, and defined the aggregate contour over all breathing phases as ITV.

A radiation oncologist contoured the GTVs, and the ITVs. Then we measured the maximal CC, AP and LR diameters of the GTV and the corresponding ITV for each patient. The differences between those maximal ITVs’ and GTVs’ CC, AP and LR diameters were defined as tumor CC, AP and LR maximal excursions, respectively. We also calculated the GTV and ITV volumes using the Eclipse^®^ planning system.

### 2.5. Tumor Excursions Measurements–Calypso^®^

The Calypso^®^ system calculates, by default, the geometric center of detected transponders, based on the initial location of each individual transponder, called the “centroid.” The centroid was set as a starting point for measurements of the tumor excursions for each patient. During SABR, Calypso^®^ tracks and measures the centroid’s excursions, corresponding to tumor movements. Patients with only two Beacon^®^ transponders implanted were eligible for measurements of all axial tumor motions. All measurements were recorded while patients were in a treatment position on the treatment table, in free breathing.

**Figure 3:**
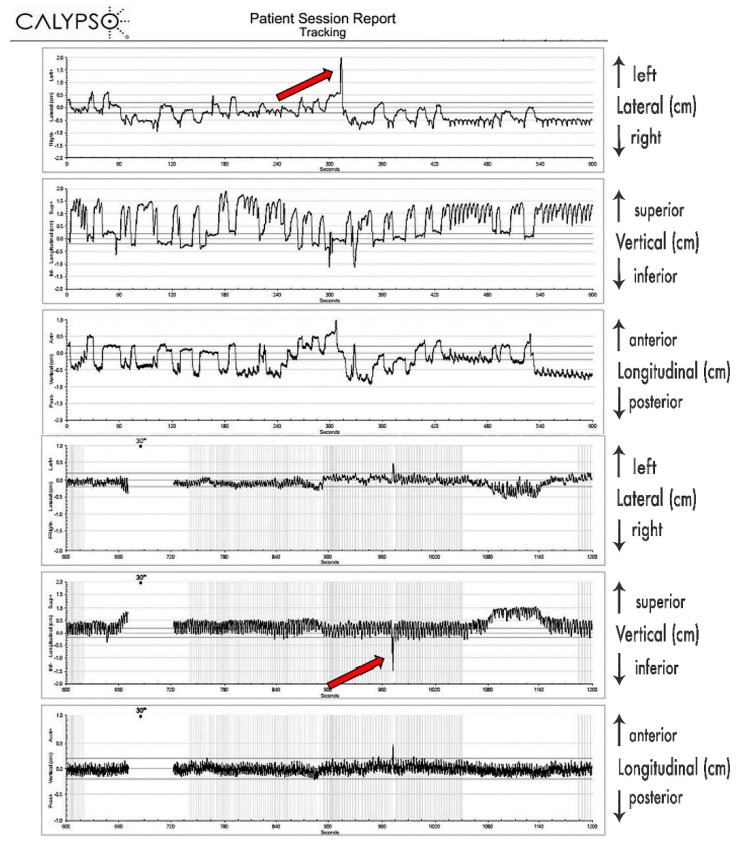
The typical Calypso^®^ “Patient Session Report” form, with the graphical presentation of the recorded fiducial excursions in the lateral, vertical and longitudinal axes, during one fraction. The isolated, short-lasting movements of the tumor on the graphical presentation are marked using red arrows.

The maximal excursions in the CC, AP and LR directions of the tumors were defined as an average of 95th percentile amplitudes of the centroids in the corresponding direction, recorded during each fraction, for each patient. This was done to avoid possible biases caused by:

1. Single extremes of centroid amplitudes.
2. Isolated, short-lasting movements (few seconds or less, spike-alike on the graphical reconstructions) (Figure 3), as they were considered to be incidental (e.g., coughing, drifts) and unsubstantial to overall movements of the tumor

### 2.6. Calypso^®^-Based ITV

For the purpose of this study, we invented and generated the Calypso^®^-based ITV (c-ITV), using the data of the Calypso^®^ Beacon transponders’ movements collected from the Calypso^®^ system. This was performed as follows: first, we loaded the Eclipse^®^’s feature “Margin to Structure” with data of previously defined maximal tumor excursions in the CC, AP and LR directions, as recorded with Calypso^®^, for each patient. The feature then expanded each GTV in the corresponding directions three-dimensionally and generated the c-ITV for each patient. The GTVs used for c-ITV generation were also the position and anatomy of the tumor on the phase-gated 4D-CT scan in the 20% inhale phase.

The purpose of the c-ITV generation was to account for virtually all possible excursions of the tumor, and to present them in the form of a target volume, similar to the standard 4D-CT based ITV.

Our assumed advantages of the c-ITV in comparison to the standard 4D-CT based ITV were:

1. Data of lesion movements were collected during a significantly longer time than during a few breathing cycles of the patient.
2. Reconstruction of this volume was based on all kinds of lesion movements, registered directly.
3. Measurement of the movements took place on the treatment table, during the actual treatment.

Using Eclipse^®^, we calculated the volume of each c-ITV for statistical comparison with the volumes of the 4D-CT generated ITVs.

### 2.7. Statistical Methods

Descriptive statistics were used to measure of central tendency and scatter (N, median, mean, standard deviation, minimum, maximum and sum). A paired samples *t*-test was used to access the level of statistical significance between values of the CC, AP and LR tumor excursions obtained with 4D-CT and Calypso^®^. A paired samples *t*-test was also used to assess the level of statistical significance between the values of the ITV and the c-ITV. A Friedman test was used for multiple comparisons between variables.

## 3. Results

**Figure 4:**
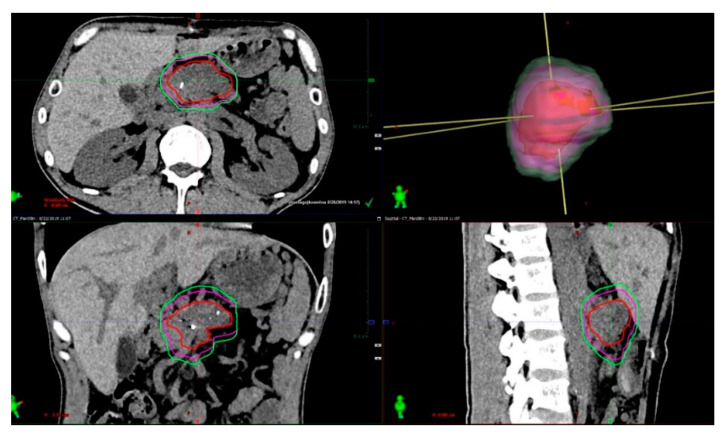
The typical ratio between the GTV (red), ITV (magenta) and c-ITV (light green), presented on MSCT in the coronar, sagittal and transversal planes, and on a 3D reconstruction.

The median (mean) volume of the GTV was 41.6 (45.7) ccm (ranging 6.4–126.1 ccm). The median (mean) volume of the c-ITV was 146.4 (160.1) ccm (ranging 45.1–346.2 ccm). The median (mean) volume of the ITV was 69.7 (78.5) ccm (ranging 12.2–176.4 ccm) (Figure 4).

**Figure 5:**
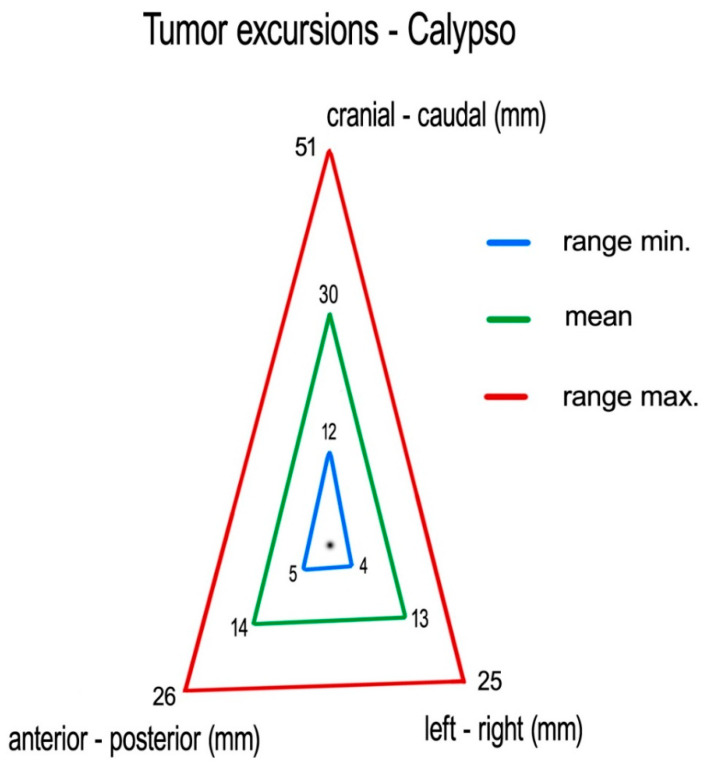
Mean tumor excursions (with range max/min), as recorded with Calypso^®^.

Measuring centroid excursions using Calypso^®^, we found the following medians (means) of tumor excursions in the corresponding directions: CC: 29 (30) mm (ranging 12–51 mm), AP: 14 (14) mm (ranging 5–26 mm) and LR: 11 (13) mm (ranging 4–25 mm) (Figure 5).

**Figure 6:**
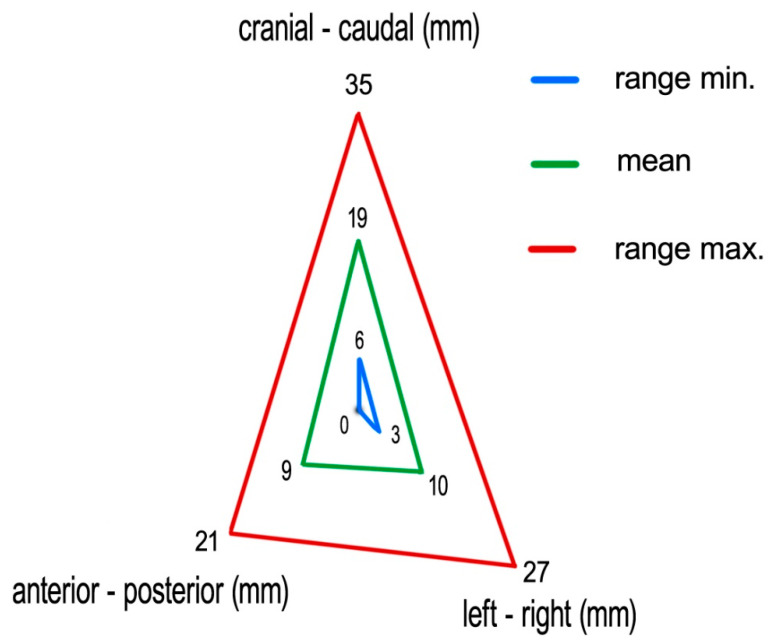
Mean tumor excursions (with range max/min) as recorded with 4D-CT.

Measuring the 4D-CT study sets, we found following medians (means) of tumor excursions in the corresponding directions: CC: 19 (19) mm (ranging 6–35 mm), AP: 9 (9) mm (ranging 0–21 mm), and LR: 9 (10) mm (ranging 3–27 mm) (Figure 6).

The differences between the Calypso^®^ and 4D-CT median recordings of maximal tumor excursion were in the CC, AP and LR directions 10 mm, 5 mm and 2 mm, respectively. The median c-ITV was 110% larger than the median ITV. The results of the descriptive statistics are presented in Table 1.

The paired sample *t*-test showed, for all tests, that the tumor excursions measured using Calypso^®^ were significantly larger than tumor’s excursions measured using 4D-CT in all directions: CC (*p* < 0.001), AP (*p* < 0.001) and LR (*p* < 0.039). The volume of the c-ITV was significantly larger than that of the ITV (*p* < 0.001). The results of the paired samples *t*-test are shown in Table 2.

On the multiple comparison Friedman test, a larger primary tumor (GTV > 45.7 ccm) occurred significantly more frequently in male patients than in female patients (*p* < 0.05), and it was also found more frequently in the pancreatic head then in the pancreatic body or tail (*p* < 0.05). Moreover, both the ITV and c-ITV were significantly larger (ITV > 78.4 ccm and c-ITV > 160.1 ccm) in male patients and in tumors located in the pancreatic head. No variable significantly affected solely the volume of the ITV or c-ITV. Location of tumor in pancreatic head was significantly more frequent in male and in older patients (*p* < 0.05). The results of Friedman test are presented in Table 3.

## 4. Discussion

This work is, to our knowledge, the first that presents the full extent of all pancreatic cancer movements during SABR using the data collected using the Calypso^®^ extracranial tracking system. Most of the studies published in recent years have reported the data of recorded pancreatic cancer movements or reductions of movements collected during respiratory motion management techniques, such as motion mitigation, deep breath hold gating, respiratory gating, or retrospective 4D-CT planning, or they have reported the data of pancreatic cancer movements recorded using fluoroscopic cone beams during fiducial tracking. Heinzerling et al., in their study from 2008, examined the motion of nearby organs during the stereotactic treatment of lung and liver tumors in 10 patients. According to their findings, abdominal compression reduced the overall motion of the pancreas by one third [25]. Lovelock et al., in 2014, analyzed the data of 42 abdominal cancer patients. Three patients had pancreatic cancer. They found, using fluoroscopic imaging, mean reduction of the target motion in the cranio-caudal direction from 11.4 mm (ranging 5–20 mm) to 4.4 mm (ranging 1–8 mm) with abdominal compression [26]. Their goal was to apply a level of the pressure sufficient to limit the CC motion of the target < 5 mm on pre-simulation fluoroscopic images. The reported average range of compressed motion in the work of Campbell WG et al. was slightly higher, 8.5 mm, probably due to differences in their procedures. In this study, no pre-simulation imaging with compression was acquired, and pressure was increased until the patient began to feel discomfort [15]. In another study on 36 pancreatic cancer patients, Huguet et al. used 4D-CT to measure target motion and simulate gating [27]. They reported following means (range) of target motion in free breathing: CC: 13 mm (±7 mm), AP: 3 mm (±2 mm), and LR: 6 mm (±3 mm). Using end-exhalation gating, they could reduce the motions by 46–60%. In a recent study, Zeng C et al. used the Varian real-time position management system to monitor breath hold, represented by the anterior–posterior displacement of an external surrogate. Even though the external markers indicated that the respiratory motion was within 3 mm in the DIBH treatment, significant residual internal target motion (based on fiducial or surgical clips implanted near or inside the target) was observed. The average range of motion was from 3.4 to 7.9 mm (SD 1.2 to 3.5 mm). For all patients, the target residual motions seemed to be random, with mean positions around their initial setup positions [28].

In the past years, MRI-guided motion management, combined with adaptive planning techniques for SABR, have been increasingly investigated. In a work by Heerkens et al. from 2014, an MRI was used to observe pancreatic tumor motion [29]. For 15 patients, means (ranges) of target motion without gating in the corresponding direction were CC: 15 mm (6–34 mm), AP: 3 mm (2–5 mm), and LR: 5 mm (1–13 mm). Simulated respiratory gating with a 50% duty cycle for 11 of those patients was sufficient to ensure total coverage using 5 mm PTV margins. In their following study from 2018, Heerkens et al. reported an average of 100% CC tumor motion, as calculated from the sagittal cine MRI, was 8.2 mm (range 2.7–23.8 mm). In the AP direction, the average 100% motion was 3.8 mm (range 0.8–12.6 mm). All measurements were performed with the application of the abdominal compression using a corset [30].

The presumed advantages of the Calypso^®^ extracranial tracking motion management technique over all techniques mentioned above are several: lesion movements are tracked directly and continuously during SABR with sub-millimeter accuracy in real time (GPS-alike); the technique performs in deep breath hold and in free breathing; there is no need to perform any motion management procedures during the planning of the SABR (such as prospective or retrospective 4D-CT planning); no motion mitigation is required; the system detects all possible motions; it is used for both patient positioning on a table and for motion management, and there is no additional ionizing irradiation for the patient, since the system uses non-ionizing radio frequencies to localize the transponders.

Our goal was to determine the total extent of the internal motions of pancreatic cancer. The results of our study indicated, in general, significantly larger pancreatic tumor maximal excursions measured with Calypso^®^ extracranial tracking than those previously observed, with medians (range) in following directions: CC: 29 mm (1.2–5.1 mm), AP: 14 mm (5–26 mm) and LR: 11 mm (4–25 mm). The results of the pancreatic tumor motions recorded with 4D-CT were approximate to those presented in previous published studies (medians): CC: 19 mm, AP: 9 mm, and LR: 9 mm. The medians of tumor excursions recorded using Calypso^®^ extracranial tracking were 53% larger in the CC direction, 56% larger in the AP direction, and 22% larger in the LR direction, compared to excursions recorded using 4D-CT, and the median Calypso^®^-based ITV was 110% larger than the median 4D-CT-based ITV.

Since 4D-CT by default records predominantly respiratory-induced movements, these differences were most probably caused by additional movements of the pancreatic tumor, most likely caused by peristalsis, heartbeat, changes of the breathing patterns (frequency, amplitude), stomach and intestine filling, drift, and possibly some other, yet undetermined causes. Their contributions seem to be significant. As presented in Figure 3, those additional tumor movements appear to be non-periodical, as well as being unrelated to respiratory movements, and their time share in overall tumor excursions remains to be further investigated, probably in its own study. As we determined the full extent of pancreatic tumor excursions only in free breathing, further investigation on the extent of those excursions should be conducted during motion mitigation and deep breath hold.

Our findings also suggest that there is a possibility that the usage of respiratory motion management or mitigation of solely respiratory movements during SABR could result in dose undercoverage of peripheral portions of the pancreatic tumor, as they could protrude out of the PTV, at some time during treatment. The clinical impact could include inferior local control, resulting in a local and/or distant relapse of the disease.

The usage of 4D-CT based ITVs leads to creation of very large PTVs that limit the tumor dose due to OAR constraints, and still could leave the possibility that the tumor dose is not delivered to the whole lesion. On the other hand, treating the c-ITV volume, as the representation of all possible ranges of tumor motion, would limit the tumor doses even more, making ablative dose delivery to the tumor unfeasible.

It is our impression that treatment of a tumor in a single phase with direct and continuous tracking of all tumor excursions could lead to usage of significantly narrower CTV-PTV margins, resulting in smaller PTV volumes that could allow for greater tumor dose escalations with better OAR sparing, and this approach could be superior to all alternative respiratory motion management or mitigation techniques.

There are some limitations of tumor delineation on contrast-free 4D-CT, and for that reason, phase gated 4D-CT scans (on which the tumor is much clearly visible) were used for delineation whenever the tumor was not clearly visible on retrospectively reconstructed 4D-CT, which was the case in the majority of the patients. Radiologists from our institution were consulted whenever there were any doubts about the tumor contours on the MSCT. Moreover, there was an additional possible cause: the larger movements of the pancreatic tumor recorded with the Calypso^®^ extracranial tracking system, while 4D-CT scans may have underestimated the real extent of the movement either because they represent only a one-day image, and respiratory motion was either not as uniform as supposed, or the method had insufficient ability to correctly outline the GTV.

## 5. Conclusions

Pancreatic tumors seem to have a significantly larger extent of full internal motion than reported in previously published studies (cited in Discussion), and those motions are most probably induced by additional causes (peristalsis, heartbeat, changes of the breathing pattern (frequency, amplitude), stomach and intestine filling, drift, and possibly some other still undetermined), besides respiration. Those additional motions should be taken into account during respiratory motion management or motion mitigating techniques for SABR, as these techniques most likely fail to track all tumor motions. Our findings suggest that only direct and continuous intrafractional tracking of all possible pancreatic tumor excursions seems to provide complete coverage of the lesion with the prescribed isodose to allow for safe tumor dose escalation. Further investigations of pancreatic tumor motions in different settings are needed.

## Figures and Tables

**Table 1:** Descriptive statistics.

Variable	GTV (ccm)	ITV (ccm)	c-ITV (ccm)	AP (mm)	c-AP (mm)	CC (mm)	c-CC (mm)	LR (mm)	c-LR (mm)
Mean	45.7	78.4	160.1	9	14	19	30	10	13
Median	41.6	69.7	146.4	9	14	19	29	9	11
Std. Deviation	29.8	39.6	86.6	44	50	7	9	5	5
Minimum	6.4	12.2	45.10	0	5	6	12	3	4
Maximum	126.1	176.4	346.2	21	26	35	51	27	25
Sum	1553.9	2667.3	5442.7	306	472	654	1004	339	428
Valid (N)	34	34	34	34	34	34	34	34	34
Missing (N)	0	0	0	0	0	0	0	0	0

GTV = gross tumor volume; ITV = internal tumor volume (on 4D CT); c-ITV = Calypso^®^-based ITV AP = anterior–posterior; c-AP = Calypso^®^-based AP; CC = cranial–caudal; c-CC = Calypso^®^-based CC; LR = left–right; c-LR = Calypso^®^-based LR.

**Table 2:** Paired samples *t*-test.

Measure 4D CT	Measure Calypso	*t*	df	*p*	Mean Difference	SD Difference
ITV	c-ITV	−8.3	33	<0.0001	−81.6	9.8
AP	c-AP	−4.5	33	<0.0001	−0.5	0.1
CC	c-CC	−5.1	33	<0.0001	−1.0	0.2
LR	c-LR	−1.8	33	0.039	−0.3	0.1

ITV = internal tumor volume; c-ITV = Calypso^®^-based ITV; AP = anterior–posterior; c-AP = Calypso^®^-based AP; CC = cranial–caudal; c-CC = Calypso^®^-based CC; LR = left–right; c-LR = Calypso^®^-based LR.

**Table 3:** Friedman test: F = 14.99; df 1 = 5; df 2 = 165; *p* < 0.00001. Multiple comparisons.

Variable	Mean Rank	Difference (*p* < 0.05) from Variable nr
(1) Gender (male)	3.7647	(2) (3) (4) (5) (6)
(2) Age (>67 years)	3.1324	(1) (6)
(3) GTV (>45.7 ccm)	3.0000	(1) (6)
(4) ITV (>78.4 ccm)	2.9853	(1) (6)
(5) c-ITV (>160.1 ccm)	3.0882	(1) (6)
(6) Location (pancreatic head)	5.0294	(1) (2) (3) (4) (5)

Minimum required difference of mean rank: 0.5796.

## Data Availability

Research data are stored in an institutional repository and will be shared upon request to the corresponding author.
